# Autonomic regulation of brown adipose tissue thermogenesis in health and disease: potential clinical applications for altering BAT thermogenesis

**DOI:** 10.3389/fnins.2014.00014

**Published:** 2014-02-07

**Authors:** Domenico Tupone, Christopher J. Madden, Shaun F. Morrison

**Affiliations:** Department of Neurological Surgery, Oregon Health and Science UniversityPortland, OR, USA

**Keywords:** brown adipose tissue, hypothermia, adenosine, hibernation, torpor, therapeutic hypothermia, fever, obesity

## Abstract

From mouse to man, brown adipose tissue (BAT) is a significant source of thermogenesis contributing to the maintenance of the body temperature homeostasis during the challenge of low environmental temperature. In rodents, BAT thermogenesis also contributes to the febrile increase in core temperature during the immune response. BAT sympathetic nerve activity controlling BAT thermogenesis is regulated by CNS neural networks which respond reflexively to thermal afferent signals from cutaneous and body core thermoreceptors, as well as to alterations in the discharge of central neurons with intrinsic thermosensitivity. Superimposed on the core thermoregulatory circuit for the activation of BAT thermogenesis, is the permissive, modulatory influence of central neural networks controlling metabolic aspects of energy homeostasis. The recent confirmation of the presence of BAT in human and its function as an energy consuming organ have stimulated interest in the potential for the pharmacological activation of BAT to reduce adiposity in the obese. In contrast, the inhibition of BAT thermogenesis could facilitate the induction of therapeutic hypothermia for fever reduction or to improve outcomes in stroke or cardiac ischemia by reducing infarct size through a lowering of metabolic oxygen demand. This review summarizes the central circuits for the autonomic control of BAT thermogenesis and highlights the potential clinical relevance of the pharmacological inhibition or activation of BAT thermogenesis.

## Introduction

The presence of uncoupling protein-1 (UCP-1) in the mitochondria of brown and beige adipocytes confers on brown adipose tissue (BAT) the unique capacity to generate heat through dissociation of the energy derived from the electron transport chain from the production of ATP. BAT thermogenesis is under the direct control of central sympathetic circuits such that the release of norepinephrine onto β 3 receptors in the membrane of brown adipocytes contributes to increased lipolysis and β-oxidation of fatty acids leading to the activation of the mitochondrial process for heat production (Cannon and Nedergaard, 2004). Cold exposure produces BAT activation, both in human (Christensen et al., 2006; Cypess et al., 2009; Nedergaard et al., 2010) and rodents (Nakamura and Morrison, 2011; Morrison et al., 2012), and exposure to a warm environment leads to a reduction in the sympathetic drive to BAT, maintaining an inhibition of thermogenesis (Nakamura and Morrison, 2010).

BAT thermogenesis requires the consumption of energy stores, initially those in the BAT lipid droplets and, with extended BAT activation, those derived from catabolism of white adipose tissue. During restricted energy availability, BAT thermogenesis and its energy expenditure are inhibited, as exemplified in the suspension of the thermogenic response to cold in hibernating animals (Cannon and Nedergaard, 2004) and during food restriction or hypoglycemia (Egawa et al., 1989; Madden, 2012). Thus, in addition to the core thermoregulatory network, BAT thermogenesis can be modulated by CNS circuits not directly involved in thermoregulation, but in regulating other aspects of overall energy homeostasis. We hypothesize that such a metabolic regulation of BAT thermogenesis plays a permissive role in determining BAT thermogenesis, potentiating, or reducing transmission through the core thermoregulatory circuit controlling BAT. In this review, we will describe the core thermoregulatory circuit controlling BAT thermogenesis in response to cold or warm exposure, as well as other CNS regions whose neurons may be modulatory or permissive for the BAT thermogenesis. Additionally, we will suggest examples in which the understanding of the circuits regulating BAT thermogenesis, and thus, the opportunities for pharmacological inhibition or activation of BAT, could be clinically relevant in pathologies such as intractable fever, obesity, or brain or myocardial ischemia.

## Core thermoregulatory circuit regulating BAT thermogenesis

**Figure 1:**
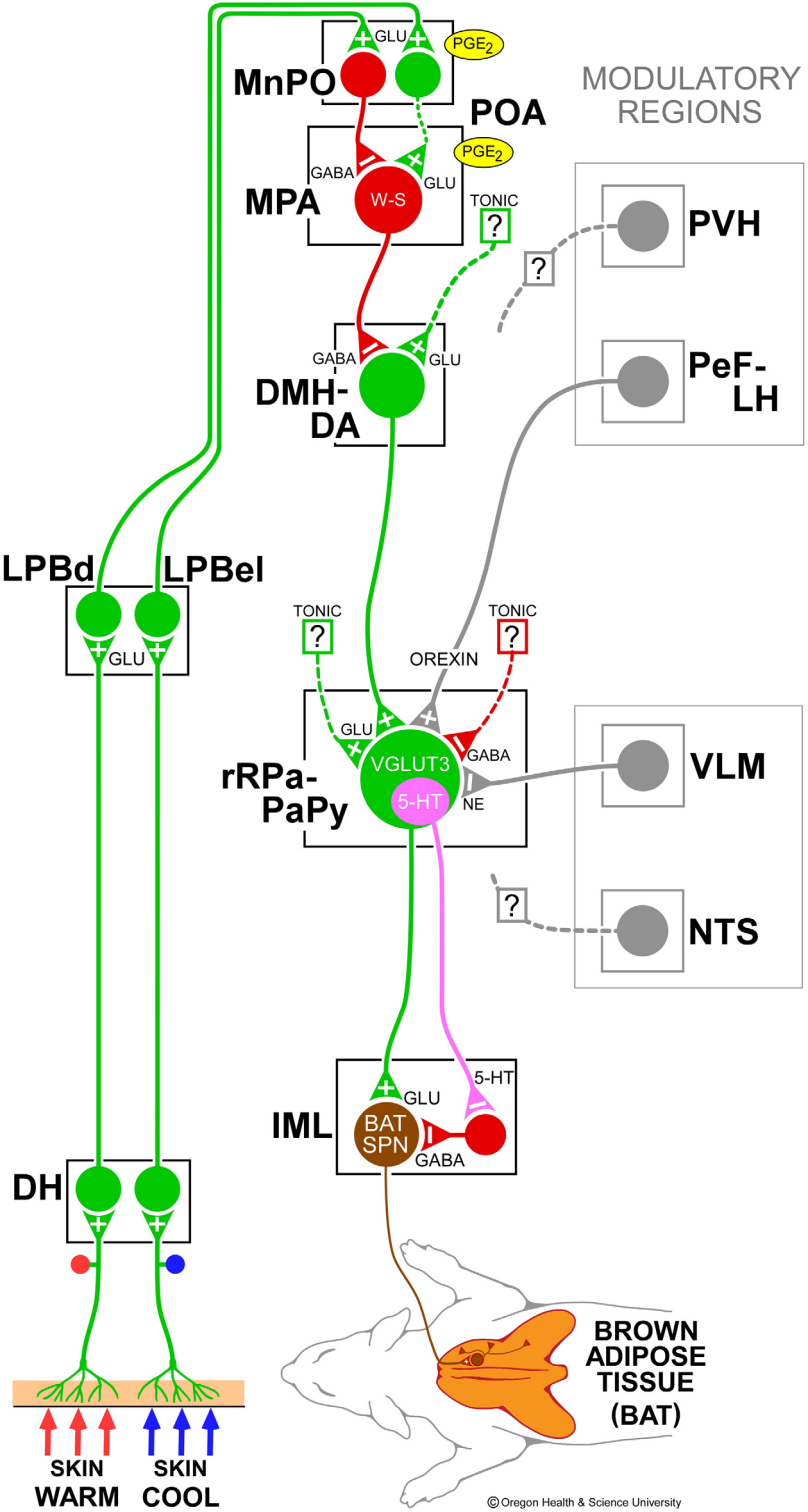
**Schematic model of the central autonomic thermoregulatory pathway and neurotransmitters regulating brown adipose tissue (BAT)**. Cool and warm cutaneous thermal sensory receptors excite primary sensory neurons in the dorsal root ganglia which relay thermal information to second-order thermal sensory neurons in the dorsal horn (DH). Cool and warm sensory neurons in DH release glutamate to activate third-order sensory neurons in the external lateral (LPBel) and dorsal (LPBd) subnuclei, respectively, of the lateral parabrachial nucleus. Thermal signals for involuntary thermoregulatory responses are transmitted from the LPB to the preoptic area (POA) which contains a population of BAT-regulating, GABAergic, warm-sensitive (W-S) neurons in the medial preoptic area (MPA) that project to inhibit glutamatergic, BAT sympathoexcitatory neurons in the dorsomedial hypothalamus and dorsal hypothalamic area (DMH-DA). In the median preoptic (MnPO) subnucleus, we postulate that GABAergic interneurons, activated by cool-activated neurons in LPBel, inhibit W-S neurons, while excitatory interneurons, excited by warm-activated neurons in LPBd, excite W-S neurons. Prostaglandin (PG) E_2_ binds to EP3 receptors to inhibit the activity of W-S neurons in the POA. The activity of BAT sympathoexcitatory neurons in the DMH-DA, determined by the balance of a glutamatergic excitation of unknown origin and a GABAergic inhibition from W-S POA neurons, excites BAT sympathetic premotor neurons in the rostral ventromedial medulla, including the rostral raphe pallidus (rRPa) and parapyramidal area (PaPy), that project to BAT sympathetic preganglionic neurons (SPN) in the spinal intermediolateral nucleus (IML). Some BAT premotor neurons can release glutamate (GLU) to excite BAT SPNs and increase BAT sympathetic nerve activity, while others can release serotonin (5-HT) to interact with 5-HT_1A_ receptors, potentially on inhibitory interneurons in the IML, to increase the BAT sympathetic outflow, and thermogenesis. Regions with modulatory inputs to the thermoregulatory pathway include the paraventricular hypothalamic nucleus (PVH) which exerts an inhibitory influence on BAT thermogenesis. Orexinergic neurons in the perifornical lateral hypothalamus (PeF-LH) project to the rRPa to increase the excitability of BAT sympathetic premotor neurons. Activation of neurons in the ventrolateral medulla (VLM) or in the nucleus of the solitary tract (NTS) produces an inhibition of BAT thermogenesis. Norepinephrine (NE) release from the rRPa terminals of VLM catecholaminergic neurons contributes to the VLM-evoked BAT sympathoinhibition via alpha 2 adrenergic receptors on BAT sympathetic premotor neurons. VGLUT3, vesicular glutamate transporter 3.

The autonomic regulation of BAT thermogenesis is effected primarily through the core thermoregulatory network (Figure 1) in the CNS. This neural network can be viewed as a reflex circuit through which changes in skin (and visceral) thermoreceptor discharge leads to alterations in the activation of BAT sympathetic nerve activity (SNA), to counter or protect against changes in the temperature of the brain and other critical organ tissues. The synaptic integration sites and neurotransmitter systems in the core thermoregulatory network constitute potential sites where non-thermal signals and pharmacological agents could modulate BAT thermogenesis.

### Cutaneous thermal receptor afferent pathway

The skin contains both cool and warm thermoreceptors (Andrew and Craig, 2001; Craig et al., 2001). The predominant cold receptors are lightly myelinated Aδ fibers, active between 10°C and 40°C and less abundant warm receptors are unmyelinated C fibers, activated between 30°C and 50°C, such that both warm and cold thermoreceptors would be active at temperatures between 30°C and 35°C (Hensel and Kenshalo, 1969). The molecular mechanisms underlying activation of cutaneous thermoreceptors reside in the transient receptor potential (TRP) family of cation channels whose conductances are temperature dependent (Pogorzala et al., 2013). TRPM8, activated by menthol and cooling is the primary candidate for the cutaneous cold receptor TRP channel (McKemy et al., 2002). BAT activity and core temperature are reduced by blockade of peripheral TRPM8 (Almeida et al., 2012) or neonatal capsaicin treatment that reduces TRPM8 mRNA in dorsal root ganglia (Yamashita et al., 2008). By virtue of their location at the interface between the environment and subcutaneous tissue, the discharge of cool and warm skin thermoreceptors will be influenced by both the ambient temperature (modulated by the degree of hairiness of the skin site) and the level of cutaneous blood flow and degree of anastomosis of the cutaneous vasculature. Thus, upon exposure to a cold environment, an increase in the discharge of skin cool thermoreceptors will be sustained by the fall in ambient temperature as well as by the reflex-evoked cutaneous vasoconstriction which reduces the flow of warm blood to the skin in order to limit heat loss.

Primary thermal somatosensory fibers deliver thermal information to lamina I neurons in the spinal (or trigeminal) dorsal horn (Craig, 2002) (Figure 1). Cold-defensive, sympathetic BAT thermogenesis is driven, not by the spinothalamocortical pathway mediating perception, localization and discrimination of cutaneous thermal stimuli, but rather by a spinoparabrachiopreoptic pathway, in which collateral axons of spinothalamic and trigeminothalamic lamina I dorsal horn neurons (Hylden et al., 1989; Li et al., 2006) activate lateral parabrachial nucleus (LPB) neurons projecting to thermoregulatory networks in the preoptic area (POA). Specifically, neurons in the external lateral subnucleus (LPBel) of the lateral parabrachial nucleus (LPB) and projecting to the median subnucleus (MnPO) of the POA are glutamatergically activated following cold exposure (Bratincsak and Palkovits, 2004; Nakamura and Morrison, 2008b), and third-order warm sensory neurons in the dorsal subnucleus (LPBd) are activated in response to skin warming (Bratincsak and Palkovits, 2004; Nakamura and Morrison, 2010). Although nociceptive inputs play only a minor role (Nakamura and Morrison, 2008b), there may be other non-thermal signals that are integrated with cutaneous thermal afferent inputs to LPB neurons in the afferent pathway contributing to regulate BAT thermogenesis.

### Hypothalamic mechanisms in the thermoregulatory control of BAT thermogenesis

Within the neural circuits regulating BAT thermogenesis, the hypothalamus, prominently including the POA and the dorsomedial hypothalamus/dorsal hypothalamic area (DMH/DA), occupies a pivotal position between the cutaneous signaling related to ambient temperature and the premotor and spinal motor pathways controlling BAT thermogenesis (Figure 1). Other hypothalamic nuclei, including the perifornical lateral hypothalamus (PeF/LH) and the paraventricular nucleus (PVH), can modulate BAT SNA (see below), but are not within the core thermoregulatory pathway.

Glutamatergic activation of MnPO neurons by their LPBel inputs is an essential step in the central mechanism for eliciting cold-defensive BAT thermogenesis. Specifically, stimulation of BAT thermogenesis by activation of LPBel neurons or by skin cooling is blocked by inhibiting neuronal activity or by antagonizing glutamate receptors in the MnPO (Nakamura and Morrison, 2008a,b). MnPO neurons receiving cutaneous cold signals from LPBel neurons also presumably receive other synaptic inputs that could influence the regulation of BAT thermogenesis by cutaneous thermal afferents. For example, tuberoinfundibular peptide of 39 residues (TIP39)-mediated activation of the parathyroid hormone 2 receptor (PTH2R) on glutamatergic terminals presynaptic to MnPO neurons projecting to DMH/DA increases core temperature, likely including a stimulation of BAT thermogenesis, and interruption of TIP39 signaling in MnPO reduces cold defense capability (Dimitrov et al., 2011). Additionally, neurons in MnPO contain receptors for leptin (Zhang et al., 2011) and for PGE_2_(Lazarus et al., 2007) that also influence the activation of BAT thermogenesis. The strong activation of BAT thermogenesis by local nanoinjections of bicuculline into MnPO (Nakamura and Morrison, 2008a) is consistent with a tonic GABAergic inhibition of skin cooling-activated neurons in MnPO.

The conceptual foundation of our current understanding of the role of the hypothalamus in normal body temperature regulation and in the elevated body temperature during fever is the discovery (Nakayama et al., 1963; Boulant and Hardy, 1974) of a class of hypothalamic neurons, perhaps concentrated in the medial preoptic area (MPA), which have intrinsic temperature sensitivity: in the absence of synaptic inputs, their discharge frequency increases as the temperature of their local environment increases. The neurophysiological mechanism underlying the thermosensitivity of warm-sensitive neurons in the POA is thought to reside in a warming-dependent facilitation of the rate of rise of a depolarizing prepotential, due to an heat-induced increase in the inactivation rate of an A-type potassium current, which shortens the intervals between action potentials and thereby increases their firing rates (Boulant, 2006). Thus, cold-defensive and febrile activation of BAT thermogenesis is postulated to occur via a disinhibitory mechanism in which MnPO neurons receiving cutaneous cool signals from LPBel neurons provide a GABAergic inhibition to warm-sensitive, GABAergic (Lundius et al., 2010) inhibitory projection neurons in the MPA (Figure 1) to reduce their tonic activity, thereby resulting in disinhibition of BAT sympathoexcitatory neurons in caudal brain regions such as DMH/DA and rostral raphe pallidus (rRPa), whose excitation increases the sympathetic outflow to BAT. Consistent with this hypothesis, increases in BAT thermogenesis evoked by skin cooling or by stimulation of MnPO neurons are reversed completely by antagonizing GABA_A_ receptors in the MPA (Nakamura and Morrison, 2008a).

The DMH/DA contains the BAT sympathoexcitatory neurons antecedent to medullary BAT sympathetic premotor neurons in rRPa (Figure 1) that are critical for the cold-defense and febrile activation of BAT thermogenesis (reviewed in Dimicco and Zaretsky, 2007). The direct activation of DMH/DA neurons by local injection of NMDA or leptin (Enriori et al., 2011) increases the sympathetic tone to BAT. Bicuculline-mediated disinhibition of DMH/DA neurons increases BAT SNA (Cao et al., 2004) and BAT thermogenesis (Zaretskaia et al., 2002), consistent with a tonically-active GABAergic input, likely from warm-sensitive POA neurons, to BAT sympathoexcitatory neurons in the DMH/DA (Figure 1) (Nakamura et al., 2005). In addition, inhibition of neurons in the DMH/DA or blockade of local glutamate receptors in the DMH/DA reverses febrile and cold-evoked excitations of BAT SNA and BAT thermogenesis (Zaretskaia et al., 2003; Madden and Morrison, 2004; Morrison et al., 2004; Nakamura et al., 2005; Nakamura and Morrison, 2007). Neurons in the DMH/DA do not project directly to BAT sympathetic preganglionic neurons, but their monosynaptic projection to the rostral ventromedial medulla (Hermann et al., 1997; Samuels et al., 2002; Nakamura et al., 2005; Yoshida et al., 2009), including the principal site of BAT sympathetic premotor neurons in the rRPa (see below), has been implicated in mediating the effects of DMH/DA neurons on BAT thermogenesis. Glutamate receptor activation in the rRPa is necessary for the increase in BAT SNA and BAT thermogenesis evoked by disinhibition of neurons in the DMH/DA (Cao and Morrison, 2006). Neurons in the DMH/DA that are retrogradely-labeled from tracer injections into the rRPa express Fos in response to BAT thermogenic stimuli such as endotoxin, cold exposure or stress (Sarkar et al., 2007; Yoshida et al., 2009; Madden, 2012) and some DMH/DA neurons that project to the rRPa receive close GABAergic appositions from neurons in the MPA (Nakamura et al., 2005).

While there is evidence suggesting a role for neurons in the periaqueductal gray (PAG) in determining the level of BAT thermogenesis, potentially by influencing the output from the DMH/DA, no consistent picture has emerged of the functional organization of the PAG influence on the sympathetic outflow to BAT. Some DMH/DA neurons projecting to the caudal PAG (cPAG) express Fos in response to cold exposure (Yoshida et al., 2005) and some neurons in the cPAG are multisynaptically-connected to BAT (Cano et al., 2003), presumably including those that project directly to the raphe (Hermann et al., 1997). Neurons in the cPAG express Fos in response to cold (Cano et al., 2003), although these may not project to the rRPa (Yoshida et al., 2009). Excitation of neurons in cPAG increases BAT temperature, but without a concomitant increase in core temperature (Chen et al., 2002), while similar excitation of neurons in the lateral and dorsolateral PAG (dl/lPAG) of conscious rats does increase core temperature, in a manner dependent on activity within the DMH (De Menezes et al., 2009). In contrast, in anesthetized and paralyzed rats, skin cooling-evoked stimulation of BAT thermogenesis was unaffected by muscimol injections into the cPAG (Nakamura and Morrison, 2007). The area of the rostral ventromedial PAG (rvmPAG) contains neurons with an inhibitory effect on BAT thermogenesis that are capable of reversing the BAT thermogenesis evoked by PGE_2_ injections into POA or by disinhibition of neurons in DMH/DA (Rathner and Morrison, 2006).

### BAT sympathetic premotor neurons in the rRPa

Within the hierarchical organization of the central thermoregulatory network, neurons in the rostral ventromedial medulla, centered in the rRPa and extending into nearby raphe magnus nucleus and over the pyramids to the parapyramidal area (PaPy) (Bamshad et al., 1999; Oldfield et al., 2002; Cano et al., 2003; Yoshida et al., 2003), play a key role as BAT sympathetic premotor neurons—providing an essential excitatory drive to BAT sympathetic preganglionic neurons (SPNs) in the intermediolateral nucleus (IML) of the thoracolumbar spinal cord, which, in turn, excite sympathetic ganglion cells innervating the BAT pads (Figure 1). BAT sympathetic premotor neurons in the rRPa respond to local application of agonists for NMDA and non-NMDA subtypes of glutamate receptors and receive a potent glutamatergic excitation (Madden and Morrison, 2003; Cao and Morrison, 2006). They also receive GABAergic inhibitory inputs, which predominate under warm conditions to reduce BAT thermogenesis. Relief of this tonically-active, GABAergic inhibition as well as an increase in glutamate-mediated excitation, including that from the DMH (Cao and Morrison, 2006), contributes to the cold-evoked and febrile increases in BAT premotor neuronal discharge that drives BAT SNA and BAT heat production (Madden and Morrison, 2003). Reduced activity of rRPa neurons produces dramatic falls in body temperature in conscious rats (Zaretsky et al., 2003). The activity of rRPa neurons is required for the increases in BAT SNA and BAT thermogenesis elicited by a variety of thermogenic stimuli, including not only skin cooling and fever (Nakamura et al., 2002; Madden and Morrison, 2003; Nakamura and Morrison, 2007; Ootsuka et al., 2008), but also disinhibition of neurons in the DMH (Cao et al., 2004) or PeF/LH (Cerri and Morrison, 2005); activation of central mu-opioid receptors (Cao and Morrison, 2005), central melanocortin receptors (Fan et al., 2007) or preoptic CRF receptors (Cerri and Morrison, 2006) and systemic administration of the adipose tissue hormone, leptin (Morrison, 2004). BAT thermogenesis is driven by the activity of both VGLUT3-expressing and serotonin-containing neurons in the rostral ventromedial medulla, as indicated by the findings that a significant percentage of VGLUT3-containing neurons in the rRPa express c-fos in response to cold exposure or icv PGE_2_ (Nakamura et al., 2004), that serotonergic neurons in the rRPa increase their firing rate in response to PGE_2_ administration or cold exposure (Martin-Cora et al., 2000), that blockade of spinal glutamatergic receptors attenuates increases in BAT SNA evoked by disinhibition of neurons in the raphe pallidus (Nakamura et al., 2004), and that blockade of spinal serotonin receptors markedly attenuates cold-evoked increases in BAT SNA (Madden and Morrison, 2010). Thus, the rRPa and PaPy regions of the ventromedial medulla contain the principal populations of BAT sympathetic premotor neurons that provide the final common medullospinal pathway (Figure 1) for the BAT sympathoexcitatory drive to the spinal network controlling BAT SNA and that are both necessary and sufficient for the BAT thermogenic responses to thermoregulatory (Figure 1) and febrile stimuli and to a variety of neurochemical mediators that influence body temperature.

### Spinal sympathetic mechanisms influencing BAT thermogenesis

The discharge of BAT SPNs that determines the level of BAT SNA and BAT thermogenesis, as well as the rhythmic bursting characteristic of BAT SNA, is governed by their supraspinal and segmental inputs as well as those to the network of spinal interneurons that influence BAT SPN excitability. A significant fraction of the BAT sympathetic premotor neurons in rRPa and in the PaPy are glutamatergic and/or serotonergic and/or GABAergic neurons (Cano et al., 2003; Nakamura et al., 2004; Stornetta et al., 2005). In addition, IML-projecting neurons located in the rRPa and the PaPy can contain thyrotropin-releasing hormone (TRH) and substance P (Sasek et al., 1990), but a role for these neurotransmitters in the spinal mechanisms regulating BAT thermogenesis has yet to be demonstrated. GABAergic and serotonergic inhibitory inputs to GABAergic spinal interneurons likely play a role in the regulation of BAT thermogenesis (Stornetta et al., 2005; Madden and Morrison, 2008). Glutamate and 5-HT play critical roles in the descending excitation of BAT sympathetic preganglionic neurons by their antecedent premotor neurons in the rRPa (Nakamura et al., 2004; Madden and Morrison, 2006, 2010). The significant role of serotonin-containing neurons in normal cold defense responses is also supported by the finding that mice that lack almost all central serotonergic neurons show blunted BAT thermogenesis during cold exposure (Hodges et al., 2008).

## Non-thermoregulatory modulation of BAT thermogenesis

The CNS circuit described above (Figure 1) represents the thermoregulatory backbone pathway controlling the BAT sympathetic outflow in response to changes in skin thermoreceptor discharge. However, BAT thermogenesis can be markedly influenced by a variety of metabolic signals (e.g., oxygen or energy status) and BAT thermogenesis can contribute to the elevations in core temperature that characterize various behavioral states (e.g., wakefulness or stress). With the view that cold-defense is the primary function of BAT thermogenesis, we propose that such influences on BAT thermogenesis are effected by modulating, perhaps in a “permissive” manner, transmission through the synaptic integration sites in the backbone thermoregulatory pathway driving BAT SNA by a diverse array of non-thermoregulatory inputs. Since it is only for the regulation of BAT thermogenesis by skin thermoreceptors that the reflex pathway from stimulus to effector has been delineated, we can only speculate about the “functional” role underlying the myriad of neurochemical and site-specific effects on BAT thermogenesis that have been described. Although we categorize these influences as “modulatory,” it should be clear that some (e.g., hypoxia or hypoglycemia) are capable of completely abrogating thermoregulatory activation of BAT thermogenesis. On the other hand, it is expected that modulatory influences that increase BAT thermogenesis (e.g., orexin) will require activation of the core thermoregulatory system.

### Orexin neurons in the PeF/LH increase BAT thermogenesis

Orexin neurons, a population of glutamatergic neurons co-expressing the peptides orexin A and B (De Lecea et al., 1998; Sakurai et al., 1998), are located exclusively in the PeF/LH and regulate a variety of physiological functions, including BAT thermogenesis, through their projections to several regions of the CNS (Peyron et al., 1998). A subpopulation of orexin neurons project to BAT sympathetic premotor neurons in the rRPa and PaPy (Oldfield et al., 2002; Berthoud et al., 2005; Tupone et al., 2011). Administration of orexin into the 4th ventricle increased c-fos expression in rRPa (Berthoud et al., 2005) and direct nanoinjection of orexin in RPa/PaPy, or activation of LH by activation of local NMDA receptors (Tupone et al., 2011) or by disinhibition with the GABA_A_ antagonist, bicuculline (Cerri and Morrison, 2005), increases BAT SNA and BAT thermogenesis. Orexin in rRPa, as well as activation of neurons in PeF-LH by NMDA, potentiates an ongoing BAT SNA but fails to increase BAT SNA if the ongoing level of BAT SNA is low, as during normothermia. These data are interpreted to indicate that the orexin input to the rRPa can amplify BAT thermoregulatory responses elicited at the level of the BAT sympathetic premotor neuron (Tupone et al., 2011). Of interest is the finding that disinhibition of PeF-LH neurons with local nanoinjection of bicuculline evokes an increase in BAT SNA even in a thermoneutral condition with an initial low level of BAT SNA and this stimulation of BAT SNA requires the activity of BAT sympathoexcitatory neurons in the DMH/DA (Cerri and Morrison, 2005). Furthermore, PeF-LH orexinergic neurons, but not their release of orexin, are required for febrile and stress-induced thermogenesis (Takahashi et al., 2013). Thus, glutamate release from orexin (and non-orexinergic) neurons in PeF-LH at projection sites of these neurons such as the DMH (Peyron et al., 1998) and the rRPa (Tupone et al., 2011; Madden, 2012) could also be an important modulator of BAT thermogenesis. The modulatory role of orexin release in rRPa on cold-defensive BAT thermogenesis (Tupone et al., 2011); the increase in body weight together with the dysregulation of body temperature observed in orexin neuron-ablated mice (Hara et al., 2001, 2005; Perez-Leighton et al., 2013); and the association between a propensity for obesity and thermoregulatory dysfunction in narcoleptic disease (Plazzi et al., 2011), a pathology characterized by the lack of the orexinergic neurons, suggests that the influence of the orexin input to the core thermoregulatory network controlling BAT SNA plays a significant role in the maintenance of thermoregulatory and metabolic homeostasis.

### Hypoxic inhibition of BAT thermogenesis

To conserve metabolic fuel reserves and oxygen for the metabolic demands of essential tissues such as the brain and heart, BAT thermogenesis is markedly influenced by the energy status of the animal: adequate fuel substrate and oxygen availability are permissive for the activation of BAT thermogenesis, while a reduced supply of nutrient fuels (Rothwell and Stock, 1982; Buchanan et al., 1991; Madden, 2012) or of oxygen (Madden and Morrison, 2005) inhibits BAT thermogenesis. Although the neural mechanisms through which metabolic homeostasis regulates the permissive control of BAT energy expenditure are only beginning to be elucidated, recent evidence supports a role for the integration of metabolic signals with the regulation of BAT thermogenesis within the nucleus tractus solitarius (NTS) (Cao et al., 2010; Grill and Hayes, 2012), the paraventricular nucleus of the hypothalamus (PVH) and the ventrolateral medulla (VLM) (Ritter et al., 2001; Cao et al., 2010; Madden, 2012).

Systemic hypoxia produces a prompt and complete reversal of the elevated BAT SNA resulting from cold exposure or PGE_2_ injection into the POA (Madden and Morrison, 2005). These effects arise from stimulation of the arterial chemoreceptors since they are eliminated by transection of the carotid sinus nerves or by inhibition of second-order arterial chemoreceptor sensory neurons in the commissural region of the nucleus of the tractus solitarius (commNTS) (Madden and Morrison, 2005). Interestingly, hypoxia also eliminates the BAT SNA activation resulting from bicuculline nanoinjection into the rRPa (Madden and Morrison, 2005), suggesting that the hypoxic inhibition of BAT thermogenesis is unlikely to arise from activation of a GABAergic input to BAT sympathetic premotor neurons in rRPa. Similarly to arterial hypoxia, disinhibition of neurons in the rostral ventrolateral medulla (rVLM) inhibits the increase in BAT SNA following nanoinjection of bicuculline into the rRPa (Cao et al., 2010). The neuroanatomical pathway for the arterial chemoreceptor-mediated inhibition of BAT SNA and BAT thermogenesis may parallel that described for the hypoxic activation of vasoconstrictor sympathetic outflow (Guyenet, 2000). Interestingly, both anatomical (Stornetta et al., 2004) and electrophysiological (Deuchars et al., 1997) studies support the existence of a bulbospinal inhibitory pathway from the rVLM to SPNs thus providing a putative descending inhibitory substrate for the hypoxic inhibition of SPNs governing BAT thermogenesis.

### Role of NTS in metabolic regulation of BAT

**Figure 2:**
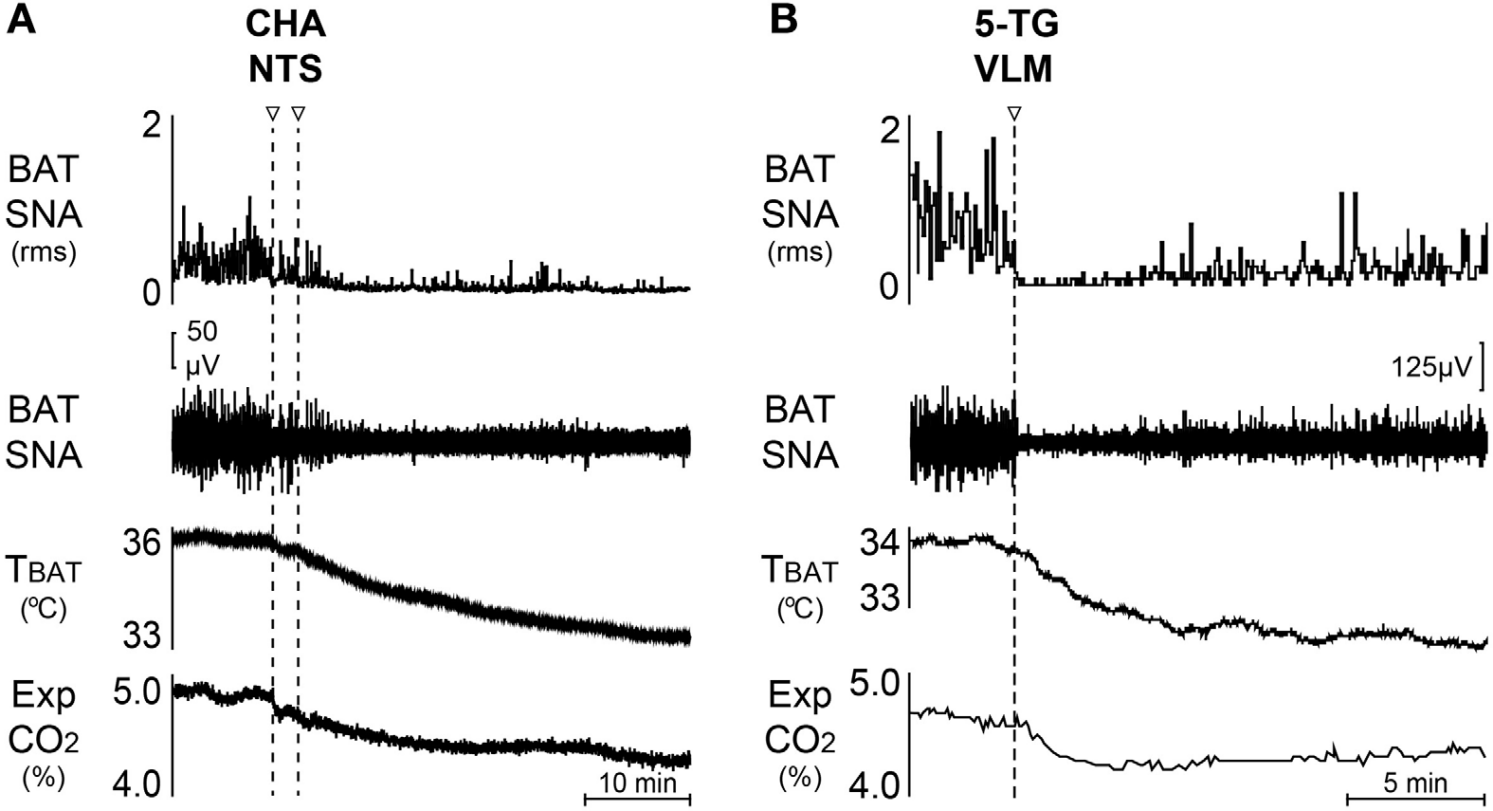
**Inhibition of BAT thermogenesis through central modulatory areas. (A)** Bilateral injection of the A1 adenosine receptor agonist, CHA, induces a rapid inhibition of the cold-evoked BAT SNA and reduces BAT temperature and expired CO_2_. Modified from Tupone et al. (2013a). **(B)** Unilateral nanoinjection of the glucoprivic agent, 5-TG, into the VLM induces a rapid inhibition of cold-evoked BAT SNA and a fall in BAT temperature and expired CO_2_. Modified from Madden (2012), Tupone et al. (2013a).

The intermediate NTS (iNTS) contains second-order sensory neurons receiving visceral vagal input that includes metabolic signals related, at least in part, to fuel substrate availability. The iNTS also contains BAT sympathoinhibitory neurons: disinhibition of iNTS neurons elicits a prompt and complete inhibition of the increases in BAT SNA and BAT thermogenesis due to cold exposure, to injections of PGE_2_ into the MPA, to disinhibition of neurons in DMH/DA or in rRPa, or to pontomedullary transection (Cao et al., 2010). Further, nanoinjection of an A1 adenosine receptor agonist in iNTS inhibits cold-evoked BAT SNA and this BAT sympathoinhibition is reversed by inhibition of iNTS neurons (Figure 2A) (Tupone et al., 2013a). The inhibition of BAT thermogenesis and BAT energy expenditure by upregulation of hepatic glucokinase may also be mediated by BAT sympathoinhibitory neurons in NTS since it is dependent on a vagal afferent input (Tsukita et al., 2012). The circuit through which iNTS neurons inhibit BAT SNA is debated and remains to be further elucidated. In the mouse, a direct GABAergic projection from NTS to BAT sympathetic premotor neurons in rRPa has been suggested to mediate the NTS-evoked inhibition of BAT activity (Kong et al., 2012). However, perhaps due to a species difference, retrograde tracing from the rat rRPa failed to identify a direct projection from iNTS to rRPa (Tupone et al., 2013a). Additionally, the long survival times necessary to transynaptically label iNTS neurons after inoculation of BAT with pseudorabies virus (Cano et al., 2003) is not consistent with a direct projection from iNTS to rRPa in rat. Moreover, activation of iNTS neurons in the rat inhibits BAT SNA and BAT thermogenesis after bicuculline injection into rRPa (Cao et al., 2010), a finding that is also inconsistent with a direct GABAergic input from the iNTS to BAT sympathetic premotor neurons in the rRPa. A species difference notwithstanding, these data could also be explained by the inability to narrowly target tracer injections into rRPa in mice and the existence of a GABAergic connection between parts of the NTS and RPa that are different from those examined in the rat. Nonetheless, the iNTS-evoked inhibition of BAT SNA in rat appears to be mediated by a multisynaptic pathway from iNTS neurons to BAT sympathetic premotor neurons in rRPa and eventually to BAT SPNs or the projection of iNTS neurons to more rostral or caudal area of the RPa. The iNTS also contains BAT sympathoexcitatory neurons, as suggested by the increase in BAT temperature following injection of leptin and/or TRH into the 4th ventricle (Hermann et al., 2006; Rogers et al., 2009), although injection of leptin alone into the NTS failed to alter BAT SNA (Mark et al., 2009). Additionally, the activation of BAT thermogenesis by duodenal lipid is dependent on cholecystokinin A receptor activation and on a vagal input to iNTS neurons (Blouet and Schwartz, 2012). Thus, multiple populations of neurons in the NTS can make significant contributions to the autonomic regulation BAT thermogenesis, particularly in response to peripheral metabolic signaling.

### Neurons in the VLM contribute to the hypoglycemic inhibition of BAT thermogenesis

Activation of neurons throughout the rostral-caudal extent of the VLM from the facial nucleus to the lateral reticular nucleus produces an inhibition of BAT SNA (Cao et al., 2010). In particular, disinhibition of rostral VLM neurons elicits a prompt and complete inhibition of BAT SNA and BAT thermogenesis elicited by cold, by injection of PGE_2_ into the MPA, by disinhibition of neurons in DMH/DA or the rRPa, or by pontomedullary transection (Cao et al., 2010). Feeding and adrenal medullary responses to the glucopenia produced by systemic administration of 2-Deoxy-D-glucose (2-DG) are mediated by neurons in the intermediate VLM, including those that project to the PVH (Ritter et al., 2001) or the spinal cord (Madden et al., 2006). Direct injection of the glucoprivic agent, 5-Thio-D-glucose (5-TG), into the intermediate VLM (Figure 2B) inhibits BAT SNA and BAT thermogenesis (Madden, 2012). Although the inhibition of BAT SNA and BAT thermogenesis from activation of iVLM neurons is mediated in part by a direct catecholaminergic projection to rRPa and dependent on α2 adrenergic receptors in rRPa (Madden et al., 2013), it's role in the glucoprivic inhibition of BAT SNA remains to be determined. In this regard, the rRPa does not receive a direct input from neurons in the rostral VLM (Madden et al., 2013), a VLM region from which potent inhibition of BAT SNA can be elicited (Cao et al., 2010), suggesting that there are multiple BAT sympathoinhibitory systems over the rostral-caudal extent of the VLM.

### Neurons in the PVH modulate BAT SNA

The PVH plays a major role in the regulation of energy homeostasis through its influence on food intake (Atasoy et al., 2012) and energy expenditure (Madden and Morrison, 2009). Although the pauci-synaptic connections of neurons in the PVH to BAT (Bamshad et al., 1999; Oldfield et al., 2002; Cano et al., 2003; Yoshida et al., 2003) strongly supports a role for these neurons in the sympathetic regulation of BAT thermogenesis, their influence on the regulation of BAT thermogenesis has been controversial. Initially, neurons in the PVH were thought to play a role in the excitation of BAT SNA, since neurons in the dorsal PVH with direct projections to the spinal SPNs are activated during fever (Zhang et al., 2000) and lesions of PVH attenuated fever (Horn et al., 1994; Caldeira et al., 1998; Lu et al., 2001), although, curiously, cold-evoked BAT thermogenesis was unaffected by lesions of the PVH (Lu et al., 2001). In contrast, disinhibition of neurons in PVH or their glutamatergic activation with NMDA injections completely inhibits BAT SNA and BAT thermogenesis induced by cold exposure, injections of PGE_2_ into the MPA, or disinhibition of neurons in DMH/DA (Madden and Morrison, 2009). Although activation of PVH neurons could attenuate the increases in BAT SNA and BAT thermogenesis evoked by injections of NMDA into the rRPa, those resulting from bicuculline injections into rRPa were unaffected by disinhibition of PVH neurons, consistent with the PVH-evoked inhibition of BAT SNA being mediated by GABA_A_ receptors in the rRPa. That neurons in the PVH provide an inhibitory influence on BAT SNA is also supported by the observations that NPY presynaptically inhibits GABA release onto PVH neurons (Cowley et al., 1999) and microinjection of NPY into the PVH decreases BAT SNA (Egawa et al., 1991). These apparent controversies in the relation of PVH neurons to BAT thermogenesis, particularly during fever, might be explained by the presence of subpopulations of PVH neurons mediating contrasting effects on BAT thermogenesis or by a role of PVH neurons during fever that involves the stimulation of other fever-supporting effector systems such as the cutaneous vasculature or hormone release.

Controversy also exists concerning the role of melanocortin receptor activation in the PVH on energy expenditure and on the activation of BAT thermogenesis. Selective rescue of melanocortin-4 receptor (MC4R) expression in neurons of the PVH (and the medial amygdala) in mice lacking expression of MC4R, failed to normalize (elevate) their oxygen consumption to wild-type levels (Balthasar et al., 2005). Based on these data it was suggested that PVH MC4Rs do not mediate the energy expenditure effects of melanocortins. In contrast, other groups have demonstrated that microinjection of melanocortin receptor agonists into the PVH increases core and BAT temperatures (Song et al., 2008; Skibicka and Grill, 2009). These effects of melanocortin receptor activation could be mediated by activation of presynaptic MC4Rs, which potentiate GABAergic inputs to PVH neurons (Cowley et al., 1999). Indeed, this explanation would reconcile such a controversy, since the rescue of MC4R in the study of Balthasar et al. would only rescue the postsynaptic MC4R in PVH neurons and not those that are located presynaptically and are potentially responsible for the effects of exogenously administered melanocortin receptor agonists. This explanation is also consistent with the existence of BAT sympathoinhibitory neurons in the PVH (Madden and Morrison, 2009). The physiological conditions which stimulate the BAT sympathoinhibitory output from the PVH are unknown, but may include hypoglycemia (Madden, 2012) and hypoxia (Madden and Morrison, 2005), as well as chronic intermittent hypoxia (Sharpe et al., 2013). Another interesting possibility is that neurons in the PVH provide a tonic inhibition of BAT thermogenesis and release from this inhibition under specific conditions, such as changes in dietary composition or leptin binding to arcuate neurons (Kong et al., 2012), may activate BAT SNA and BAT energy expenditure.

## Pathology

### BAT thermogenesis contributes to fever

**Figure 3:**
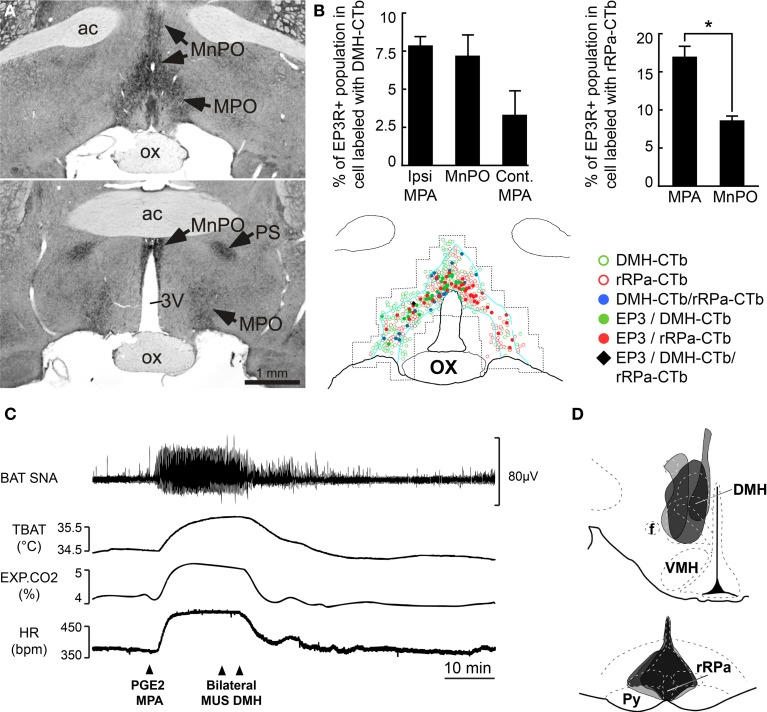
**Role of POA neurons in the generation of the fever response mediated by PGE_2_. (A)** Intense immunoreactivity for the EP3-R in the rat POA is particularly distributed in the MnPO, MPA, and parastrial nucleus (PS). Modified from Nakamura et al. (1999). **(B)** Counting of double-labeled neurons in MnPO and MPA that were EP3-R immunoreactive and retrogradely labeled following injection (**D**) of Alexa488-conjugated CTb and Alexa594-conjugated CTb in DMH and in rRPa, respectively, thereby suggesting their role in mediating the febrile response. ^*^P < 0.05, paired *t*-test. Modified from Nakamura et al. (2009). **(C)** PGE_2_-evoked increases in BAT SNA, BAT temperature (TBAT), Exp CO_2_, and HR were immediately reversed by bilateral muscimol injections into the DMH. Modified from Madden and Morrison (2004).

Fever is a hyperthermia (i.e., increase in core temperature) mediated by increased thermogenesis and cutaneous vasoconstriction in response to inflammatory mediators that influence central thermoregulatory circuits. Inflammatory mediators such as interleukin (IL)-1 (Rothwell, 1989), macrophage inflammatory protein-1 (MIP-1) (Zampronio et al., 1994) and tumor necrosis factor alpha (TNF-α) (Rothwell, 1988) are secreted in response to invading pathogens. With the exception of MIP-1 and IL-8, the febrile response to these inflammatory mediators requires the production of prostaglandin E_2_ (PGE_2_). BAT thermogenesis contributes significantly to the heat production necessary to raise core body temperature during the febrile response driven by the increased synthesis of prostaglandin E_2_ (PGE_2_) in response to pathogen invasion. PGE_2_, which is synthesized in peripheral tissues and in the brain vasculature in response to immune signals (Elmquist et al., 1997; Matsumura et al., 1998; Yamagata et al., 2001), acts through its EP3 receptor (EP3-R) on neurons in POA, particularly the MPO and MnPO (Scammell et al., 1996; Nakamura et al., 2000, 2002; Lazarus et al., 2007) to activate BAT thermogenesis and increase body temperature during fever. The central role played by the POA neurons in fever is highlighted by the demonstration that elimination of EP3-R selectively in the POA is sufficient to prevent lipopolysaccharide (LPS) fever (Lazarus et al., 2007). However, EP3 receptors expressed in other brain areas such as the PVH and parabrachial nucleus may play a minor role in the generation of fever, since a thermogenic response follows nanoinjection of PGE_2_ into these regions (Skibicka et al., 2011). Anatomical evidence supporting the role of POA neurons in fever includes the demonstration of a population of EP3-R- positive, PRV-infected neurons in POA following virus inoculation of interscapular BAT (Yoshida et al., 2003). Also, EP3-R-positive neurons in POA heavily and directly project to DMH and to rRPa (Nakamura et al., 2002, 2005, 2009) (Figures 3A,B,D). Moreover, the majority of EP3-R-expressing POA neurons (Nakamura et al., 2002) and of warm-sensitive neurons in POA (Lundius et al., 2010) are GABAergic. Physiologically, the muscimol-evoked inhibition of POA neurons elicits hyperthermic, cardiovascular, and neuroendocrine responses similar to those evoked by a PGE_2_ nanoinjection into the same site (Zaretsky et al., 2006). Consistent with these results, a fever like response is elicited by the bicuculline-evoked disinhibition of DMH neurons (Morrison, 1999; Zaretskaia et al., 2002; Cao et al., 2004). Currently, the febrile response is postulated to arise from PGE_2_ binding to EP3-R and inhibiting, via inhibitory GTP-binding proteins (Narumiya et al., 1999), the activity of warm-sensitive neurons in POA. This results in the disinhibition of DMH BAT sympathoexcitatory neurons projecting to BAT sympathetic premotor neurons rRPa, and the activation of BAT thermogenesis (Figure 3C).

### The role of BAT in obesity

**Figure 4:**
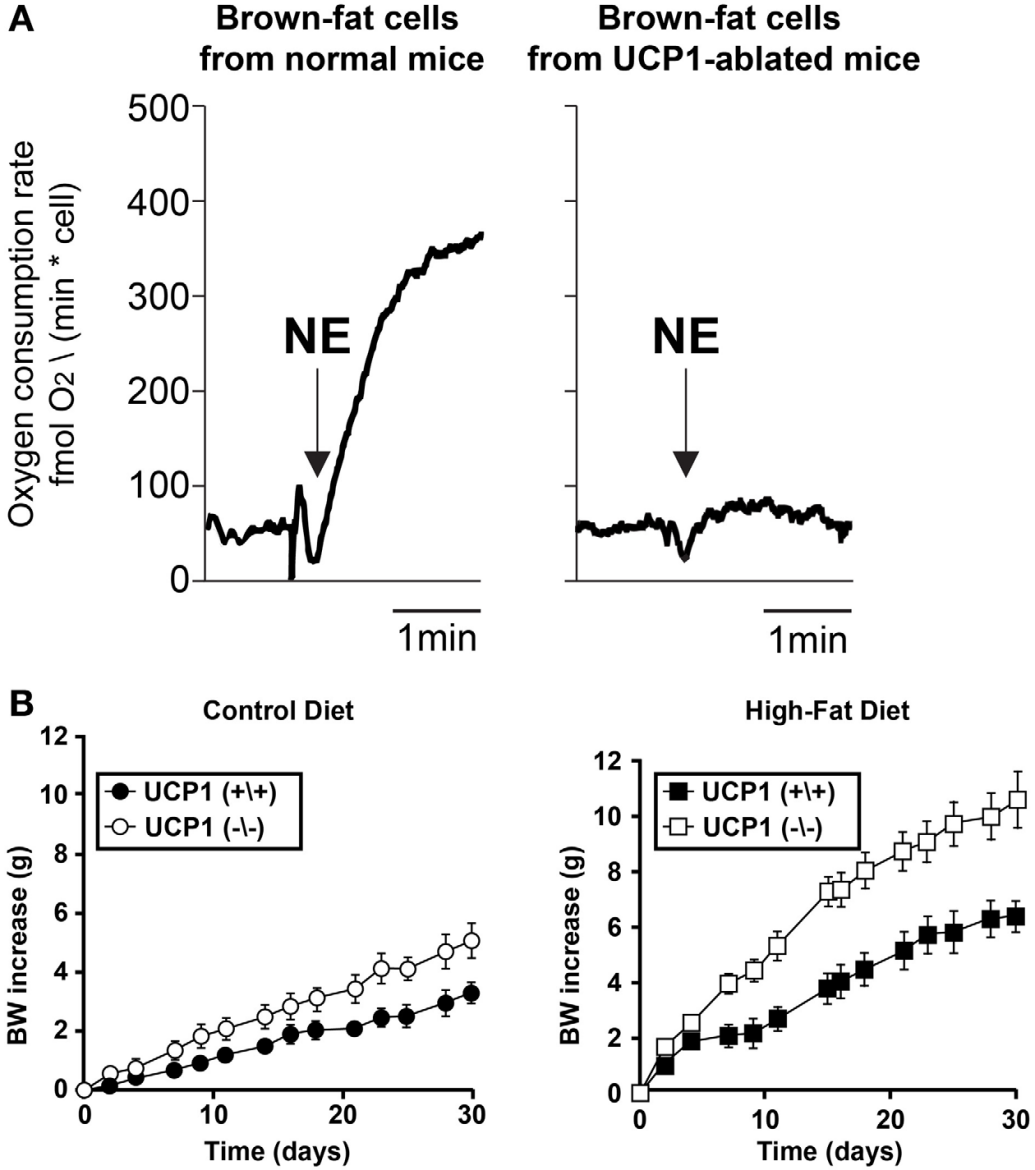
**Impairment of BAT thermogenic function leads to body weight increase**. **(A)** Brown fat cells isolated from normal mice or from UCP1-ablated mice were stimulated with 1 μM norepinephrine (NE). Thermogenic responses (oxygen consumption) were impaired in the UCP1-abated mice compared to wild type. Adapted from Matthias et al. (2000). **(B)** Body weight (BW) increase of wild-type and UCP1(−/−) mice. Average slope was significantly different (*p* < 0.05) between both wild-type and UCP1-ablated mice for control diet and high-fat diet. From Feldmann et al. (2009).

Sympathetic activation of BAT increases lipolysis and β-oxidation of fatty acids in BAT, allowing heat production, via mitochondrial UCP1, at the expense of stored lipids (Cannon and Nedergaard, 2004). Reduced thermogenesis, and thus reduced lipid consumption, in BAT may contribute to the etiology of some forms of obesity. Indeed, humans with low body temperature, suggesting a reduced thermogenesis, are more prone to obesity (Rising et al., 1995; Van Marken Lichtenbelt and Daanen, 2003) and obesity in humans is correlated with decreased BAT activity (Oberkofler et al., 1997; Rousseau et al., 2006; Van Marken Lichtenbelt et al., 2009). Furthermore, treatments that impair BAT thermogenesis (Figure 4A), such as ablation of the tissue itself or deletion of UCP-1 or β-adrenergic receptors, render rodents prone to excess weight gain (Figure 4B) (Lowell et al., 1993; Hamann et al., 1996; Bachman et al., 2002; Kontani et al., 2005; Feldmann et al., 2009). Conversely, increased BAT activity is protective against obesity (Kopecky et al., 1995, 1996; Guerra et al., 1998; Stanford et al., 2013). Regardless of the specific role that decreased expression or activation of BAT has in the development or maintenance of obesity in humans, it is clear that adult humans possess BAT (Cypess et al., 2009; Saito et al., 2009; Van Marken Lichtenbelt et al., 2009; Virtanen et al., 2009; Zingaretti et al., 2009) and that sympathetic activation of this tissue regulates the metabolism of fat in this tissue. Therefore, a greater understanding of the sympathetic regulation of BAT could suggest targets for therapeutic approaches to increase energy expenditure in this tissue and thereby combat obesity.

### Clinical relevance of BAT inhibition

**Figure 5:**
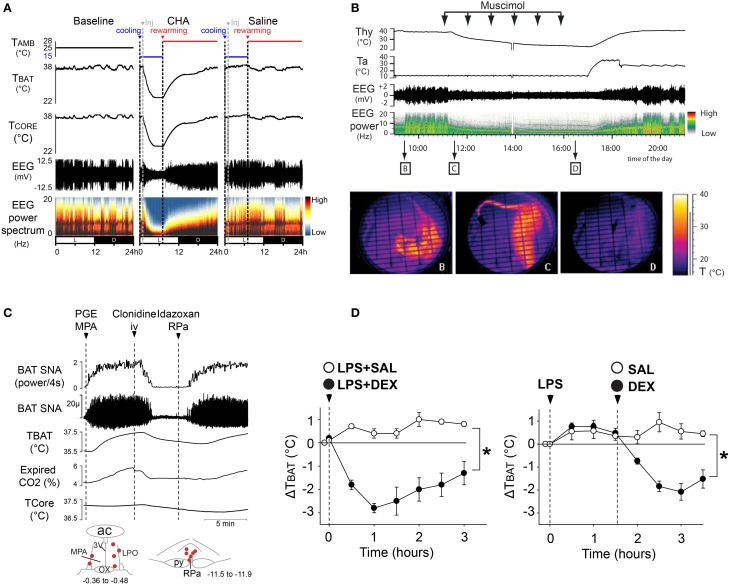
**Inhibition of BAT thermogenesis could be used to induce therapeutic hypothermia or to treat fever. (A)** Central activation of the A1 adenosine receptor (A1AR), induces a deep hypothermia and reduction of EEG amplitude and power, characteristic of a torpor-like state in rat, a non-hibernating species. External re-warming reversed the hypothermic torpor-like state, allowing recovery from this state with no apparent dysfunction in physiological and sleep characteristics. Adapted from Tupone et al. (2013a). **(B)** The inhibition of thermogenesis following administration of GABA_A_ agonist, muscimol, into the rRPa produced a deep hypothermia and reduction in EEG amplitude and a shift of the theta power resembling the torpor-like state of hibernating mammals. Adapted from Cerri et al. (2013). **(C)** Alpha2 adrenergic receptor agonist, clonidine, inhibits PGE_2_-evoked BAT SNA that is reversed by direct injection of α2 receptor antagonist in rRPa. **(D)** Alpha2 receptor agonist treatment blocks the febrile response elicited by LPS injection in a free-behaving rat. The asterisk indicates two-way repeated measures ANOVA: drug effect, *p* 0.001; time effect, *p* 0.001; and interaction effect, *p* 0.001. Adapted from Madden et al. (2013).

Although BAT is activated during human cold defense (Christensen et al., 2006), its role in human febrile thermogenesis has not been directly demonstrated. Nonetheless, since the central thermoregulatory pathways for cold-defensive and febrile thermogenesis are overlapping in rats (Nakamura and Morrison, 2011), it is highly likely that BAT thermogenesis is recruited in human fever as well. Thus, a potentially significant role for a pharmacological inhibition of BAT thermogenesis could be the inhibition of potentially lethal febrile responses, especially those resistant to treatment with COX inhibitors, such as in malaria, head trauma (neurogenic fever), meningitis, or AIDS. Although not a lethal febrile response, LPS-induced fever was reversed and prevented by central inhibition of BAT (and shivering) thermogenesis following systemic delivery of an agonist for the alpha2 adrenergic receptor (Figures 5C,D) (Madden et al., 2013), which is present in the rRPa and leads to inhibition of the activity of BAT sympathetic premotor neurons and a fall in BAT thermogenesis (Madden et al., 2013). Additionally, febrile responses were reversed by treatment with an A1 adenosine receptor agonist (Muzzi et al., 2012), which inhibits BAT thermogenesis (Tupone et al., 2013b).

Another important role for pharmacological inhibition BAT thermogenesis is the facilitation of a reduction in body temperature for therapeutic use in patients with brain or cardiac ischemia. Although hypothermia can be protective in the settings of myocardial infarction and brain ischemia (Hemmen and Lyden, 2009), the hypothermia is often induced by the use of cooling approaches (Schwartz et al., 2012) which also elicit a thermoregulatory response including BAT and shivering thermogenesis (Nakamura and Morrison, 2008b, 2011), thereby preventing a rapid and deep cooling of the body. Since BAT plays a role in the human thermogenic response during cold exposure, the pharmacological inhibition of BAT thermogenesis could contribute to a more rapid and controlled body core cooling for therapeutic hypothermia (Tupone et al., 2013b). Thus, understanding the central circuits controlling BAT thermogenesis is of fundamental importance for the development of drugs to induce hypothermia. For instance, the neural circuit described above shows several CNS sites and some of the pharmacological agents acting on specific thermoregulatory areas through which inhibition of BAT thermogenesis could be obtained. However, to be therapeutically useful, a pharmacologically-induced inhibition of thermoregulation and the associated hypothermia should not interfere with other important physiological functions and should be easily reversible. In this regard, the injection of muscimol in rRPa (Cerri et al., 2013) or the central administration of an A1 adenosine receptor agonist (Tupone et al., 2013a) inhibited BAT SNA and BAT thermogenesis in rat, which, in a cool ambient temperature, led to a deep hypothermia and hypometabolic state (Figures 5A,B) which also characterizes torpor, from which rats recovered spontaneously with no apparent physiological dysfunction. This demonstrates the possibility to produce a safe, hypothermic, and torpid state in a nonhibernating animal. We suggest that a pharmacological inhibition of BAT thermogenesis could be clinically useful in human for the rapid induction of therapeutic hypothermia or as an alternative antipyretic.

## Summary

BAT thermogenesis is finely controlled by the CNS. Cold and warm input from the skin are received in the parabrachial nuclei of the brain stem and transmitted to the POA, a center for the integration of the thermal information. Neurons in the POA provide an inhibitory regulation of BAT activation through a serial neuronal network including the DMH and the rRPa excitatory projection to the spinal sympathetic preganglionic neurons, to maintain the temperature homeostasis of the body in response to changes in the ambient temperature. However, the regulation of BAT thermogenesis is also directly related to overall energetic status. As described here, robust metabolic signals such as hypoxia and hypoglycemia inhibit BAT thermogenesis via neurons in the NTS, PVH or VLM. It is likely that these brain regions, which are also involved in the control of energy homeostasis, can exert more subtle inhibitory effects on BAT activation that are reflective of a permissive metabolic control of BAT thermogenesis. In this regard, malfunction of those metabolic controllers could result in chronic downregulation of BAT activity and BAT thermogenesis which could contribute to metabolic pathologies such as obesity and diabetes. On the other hand, it may be possible, with pharmacological stimulation of BAT thermogenesis in obese patients, to increase the energy expenditure to reduce body weight. Additionally, a better comprehension of the inhibitory regulation of BAT thermogenesis, could contribute to the discovery of novel pharmacological approaches to block cold-defensive BAT thermogenesis, which would be useful to induce therapeutic hypothermia or to treat intractable fevers. Centrally-acting drugs interacting with the A1 adenosine receptor or with the alpha2 adrenergic receptor may be applicable for such therapeutic approaches. In conclusion, control of the autonomic regulation of BAT thermogenesis, primarily a thermoregulatory function, could play a significant role in ameliorating pathologies like obesity or high fevers, or for the induction of a therapeutic hypothermic state following myocardial infarction or stroke.

### Conflict of interest statement

The authors declare that the research was conducted in the absence of any commercial or financial relationships that could be construed as a potential conflict of interest.
